# P313: Prehospital care: analysis of serological status and vaccination coverage for Hepatitis B on injured workers

**DOI:** 10.1186/2047-2994-2-S1-P313

**Published:** 2013-06-20

**Authors:** MHRS Paiva, ACD Oliveira, AOD Paula

**Affiliations:** 1 Universidade Federal de Minas Gerais, Belo Horizonte / Minas Gerais, Brazil

## Introduction

Immunization of health professionals is critical to the prevention of various diseases and is one of the factors associated with reduced mortality from these diseases.

## Objectives

The aim was to determine the prevalence of accidents involving exposure to biological material and the vaccinal and serological situation for Hepatitis B and the immunization against tetanus and diphtheria of injured professionals of the mobile emergency medical services of the State of Minas Gerais.

## Methods

An epidemiological study with a cross-sectional design was carried out, involving professionals from the Emergency Medical Services in the State of Minas Gerais. Data were collected between December 2011 and July 2012, using a structured questionnaire, and then typed and analyzed in SPSS statistical software, version 18.0. Characterization of population, vaccination coverage for tetanus, diphtheria, Hepatitis B and serological status for Hepatitis B was verified through calculation of absolute and relative frequencies.

## Results

The prevalence of accidents involving biological material was 17.0% (83/487). Vaccinal coverage for Hepatitis B, among injured professionals was 96.5%, of whom 66.1% confirmed that they had had three doses of this immunization, 41.9% confirmed that they had done the anti-HB test, with 25.7% stating that they were reactive, 9.8% non-reactive, and 64.5% stating that they did not know. The vaccinal coverage for tetanus and diphtheria reached 96.3% with 75.8% informing that the last reinforcing dose had been administered less than ten years previously.

## Conclusion

Based on these results, it is suggested that workplaces evaluated promote an adequate vaccination coverage and monitoring of vaccination and serological status of these workers.

## Disclosure of interest

None declared.

